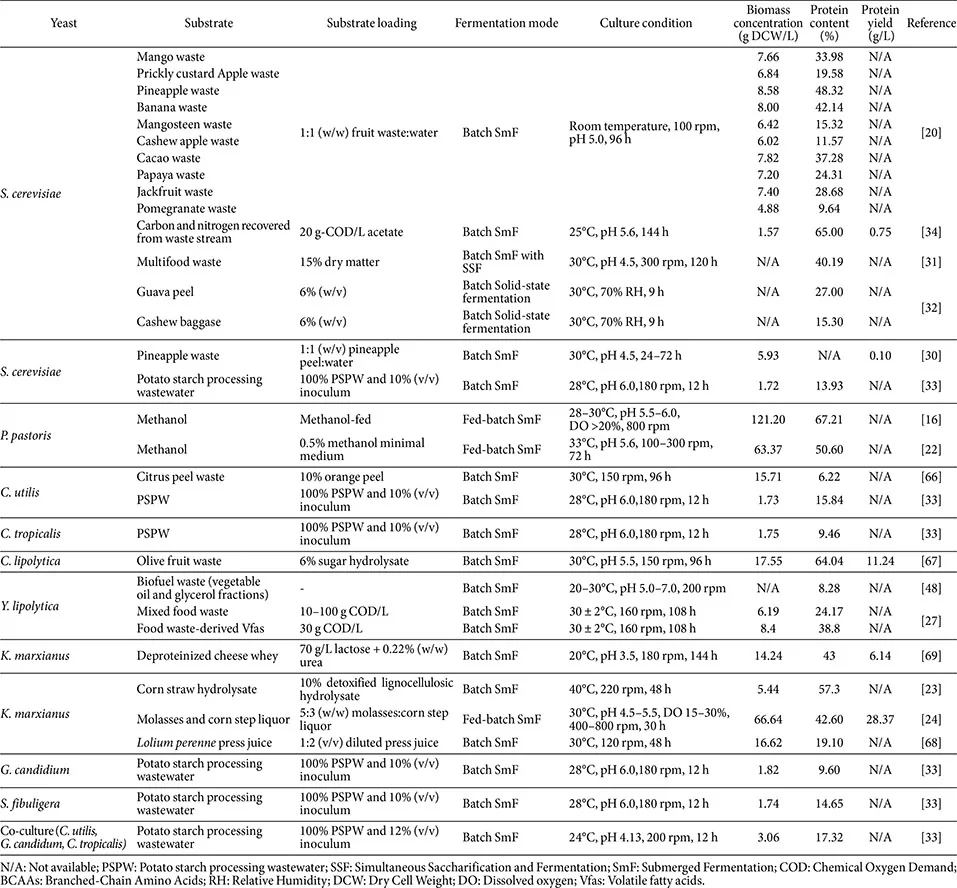# Recent Advances in Sustainable Yeast-Based Single-Cell Protein Production from Renewable Substrates Using Engineering Approaches

**DOI:** 10.4014/jmb.2607.07033

**Published:** 2026-09-02

**Authors:** Ely Yosriah, Grace Evelina, Dian Shofinita, Jeong Jae Lee, Soo Rin Kim

**Affiliations:** 1 School of Food Science and Biotechnology, Kyungpook National University, Daegu 41566, Republic of Korea; 2 Department of Food Engineering, Faculty of Industrial Technology, Institut Teknologi Bandung, Sumedang 45363, Indonesia; 3 Department of Chemical Engineering, Faculty of Industrial Technology, Institut Teknologi Bandung, Bandung 40132, Indonesia; 4 Institute for Bio Technology Convergence, Kyungpook National University, Daegu 41566, Republic of Korea; 5 Department of Advanced Bioconvergence, Kyungpook National University, Daegu 41566, Republic of Korea; 6 Research Institute of Tailored Food Technology, Kyungpook National University, Daegu 41566, Republic of Korea

**Keywords:** Yeast, Single cell protein, Metabolic engineering, Evolutionary engineering, Microbial protein, Waste valorization

## Abstract

Yeast-based single-cell protein (SCP) has emerged as a promising sustainable protein source due to its high protein content, favorable nutritional profile, and ability to utilize renewable feedstocks. The conversion of food-processing wastes, agricultural by-products, and one-carbon substrates into protein-rich biomass supports waste valorization and circular bioeconomy strategies. However, efficient utilization of these diverse feedstocks remains challenging due to variations in substrate composition and microbial metabolic capacity. Recent advances in metabolic engineering, cell wall engineering, adaptive laboratory evolution, random mutagenesis, and fermentation optimization have improved substrate utilization, biomass formation, and protein accumulation in yeasts. These developments have expanded the potential applications of yeast-based SCP in protein supplements, meat products, flavoring agents, and animal feed. This review provides a comprehensive overview of yeast species for SCP production, renewable feedstock utilization, engineering approaches for enhancing biomass and protein accumulation, and the emerging applications of yeast-based SCP in sustainable food and feed systems.

## Introduction

The global demand for protein is expected to increase substantially in the coming decades due to population growth, urbanization, rising incomes, and dietary shifts toward protein-rich foods [1]. Although conventional livestock production currently supplies most dietary protein worldwide, it requires extensive land and water resources and contributes significantly to greenhouse gas emissions and environmental degradation [2-4]. Consequently, the development of sustainable and resource-efficient alternative protein sources has become increasingly important for ensuring future food security.

As a promising alternative protein source, microbial single-cell protein (SCP) has attracted considerable attention because of its high protein content, rapid production rate, and relatively low environmental footprint compared with conventional animal-based protein sources [2, 3, 5]. SCP can be produced from microorganisms such as bacteria, fungi, algae, and yeasts using a wide range of renewable substrates, including food-processing residues, agricultural by-products, industrial waste streams, and one-carbon compounds [2, 3, 5, 6]. This ability supports waste valorization and circular bioeconomy strategies by converting low-value resources into protein-rich biomass [5, 7].

Among SCP-producing microorganisms, yeasts are particularly attractive due to their favorable nutritional profile, robust growth, long history of safe use, and remarkable metabolic versatility [8, 9]. Species such as *Saccharomyces cerevisiae*, *Pichia pastoris*, *Kluyveromyces marxianus*, *Candida utilis*, and *Yarrowia lipolytica* can efficiently utilize diverse feedstocks while producing biomass rich in protein, essential amino acids, vitamins, and bioactive compounds [6, 8-11]. However, efficient conversion of renewable substrates into protein-rich biomass remains challenging because of feedstock heterogeneity, nutrient imbalances, inhibitory compounds, and the complex regulation of carbon and nitrogen allocation within the cell [6, 7, 12, 13].

To overcome these limitations, various engineering approaches have been developed to improve SCP production in yeasts. Advances in metabolic engineering, cell wall engineering, transporter engineering, adaptive laboratory evolution, random mutagenesis, and fermentation optimization have been used to improve substrate utilization, biomass productivity, stress tolerance, and protein accumulation [7, 14-19]. These engineering approaches can support the utilization of renewable substrates, enhance tolerance to substrate-related stresses, and balance carbon and nitrogen allocation between biomass and protein synthesis. This is particularly important because variations in the composition and nutrient availability of renewable feedstocks can limit their efficient conversion into protein-rich biomass. While previous reviews have mainly discussed SCP sources, feedstocks, nutritional properties, and applications [15-18], this review focuses on genetic, evolutionary, and process-based strategies to address these challenges. In addition, the applications of yeast-derived SCP in food and feed systems are discussed, along with current limitations and future directions for sustainable production.

## Yeast as a Platform for SCP Production

The performance of yeast-based SCP production is largely influenced by the characteristics of the yeast host and the feedstock utilized. Different yeast species exhibit distinct metabolic capabilities, substrate utilization profiles, and protein-producing capacities, resulting in considerable variation in biomass productivity and SCP content [11, 12]. Likewise, feedstock composition affects nutrient availability, microbial growth, and protein accumulation during cultivation [6]. Therefore, understanding the characteristics of yeast species and their ability to utilize diverse renewable substrates is essential for developing efficient and sustainable SCP production processes.

### Major Yeast Species for SCP Production

Yeasts differ considerably in their physiological characteristics, substrate utilization capabilities, and protein-producing capacities, making host selection a key factor in SCP production. Among the available platforms, *S. cerevisiae* remains the most extensively utilized species owing to its long history of industrial use, GRAS status, and well-established genetic and fermentation toolkits [6, 20]. As summarized in Table 1, *S. cerevisiae* exhibited substantial variation in biomass concentration and protein content across different substrates and cultivation conditions. Notably, acetate-based cultivation resulted in a relatively low biomass concentration (1.57 g DCW/L) but the highest protein content (65.0%), whereas several fruit-based substrates produced higher biomass concentrations with lower protein contents. This variation indicates that biomass formation and cellular protein accumulation do not necessarily respond in parallel, as substrate composition and cultivation conditions can differently influence cell growth and protein synthesis [20].

Among the species evaluated, *P. pastoris* exhibited protein contents ranging from 50.60% to 67.21%, while biomass concentrations reached 121.2 g DCW/L under the reported cultivation conditions. Its efficient methanol assimilation and ability to achieve exceptionally high cell densities during fed-batch cultivation make it particularly attractive for SCP production from one-carbon feedstocks and industrial-scale processes. Furthermore, recent studies have demonstrated that *P. pastoris* can be effectively engineered to enhance methanol conversion efficiency, biomass formation, and protein accumulation through cell wall engineering and metabolic engineering approaches [16, 21, 22].

*K. marxianus* has emerged as a promising platform for SCP production due to its rapid growth, thermotolerance, and broad substrate utilization spectrum, including lactose, inulin, and lignocellulose-derived sugars [23-26]. These characteristics enable the efficient conversion of dairy-processing by-products and agricultural residues into protein-rich biomass, supporting the valorization of low-cost renewable feedstocks. Protein contents ranging from 19.10% to 57.30% have been reported across diverse substrates, demonstrating the strong adaptability of *K. marxianus* to different cultivation conditions and feedstock compositions.

Another promising platform is *Y. lipolytica*, an oleaginous yeast with a unique capacity to utilize hydrophobic substrates, including waste oils, lipid-rich residues, glycerol-containing biofuel wastes, and volatile fatty acids [27]. This metabolic versatility enables the efficient conversion of diverse renewable feedstocks into protein-rich biomass, particularly lipid-rich industrial wastes. Reported SCP contents ranged from 8.28% to 64.04%, indicating that protein production is strongly influenced by substrate characteristics and cultivation conditions.

*C. utilis* is a well-established platform for SCP production owing to its rapid growth, high protein content, and ability to utilize a wide range of carbon sources, including glucose, sucrose, and agro-industrial by-products [28]. Among these substrates, fruit processing by-products have attracted increasing attention as low-cost feedstocks for sustainable SCP production. Using orange peel waste and potato starch processing wastewater (PSPW), protein content ranging from 6.22% to 15.84% have been reported, demonstrating the feasibility of converting these residues into protein-rich biomass. The variation in SCP content suggests that substrate composition and cultivation conditions play important roles in determining protein accumulation.

### Substrate Versatility and Utilization of Renewable Feedstocks

Feedstock selection is a critical factor in SCP production because substrate composition directly influences microbial growth, nutrient assimilation, biomass formation, and protein accumulation. Utilization of renewable feedstocks has gained increasing attention as a strategy to reduce production costs while simultaneously valorizing waste streams generated from food processing and agricultural activities. Various organic residues contain carbohydrates, nitrogen sources, vitamins, and minerals that can support microbial metabolism and SCP synthesis. Consequently, food-processing wastes, agricultural and agro-industrial byproducts, and one-carbon compounds have been extensively investigated as alternative substrates for yeast-based SCP production. Substantial variation in SCP productivity was observed among feedstock categories summarized in Table 1.

**Food-processing wastes.** Food-processing wastes have been widely explored as feedstocks for SCP production because they are generated in large quantities and can be converted into value-added products through microbial fermentation. The substrates evaluated in this category included pineapple waste, papaya waste, cashew apple waste, mango waste, jackfruit waste, cacao waste, prickly custard waste, banana waste, mangosteen waste, pomegranate waste, and food waste. Most studies employed submerged fermentation at temperatures ranging from 25°C to 30°C and pH values between 4.5 and 5.6, indicating that SCP production was generally conducted under relatively similar cultivation conditions. Fermentation durations ranged from 56 h to 144 h depending on the feedstock and cultivation strategy applied. Despite these similarities, substantial differences in SCP production were reported among the evaluated substrates. This variation indicates that feedstock characteristics, together with differences in cultivation conditions, contributed to the observed SCP production performance [20].

Moreover, different SCP production levels were observed among food-processing wastes even when cultivation was performed under comparable operating conditions. For example, pineapple waste produced 25.65% crude protein at a pineapple peel-to-water ratio of 1:1 (w/v), whereas a ratio of 1:2 (v/v) increased crude protein content to 60.80%. This comparison demonstrates that cultivation conditions, particularly substrate loading, can substantially influence crude protein content even when the same feedstock is used. These findings demonstrate the combined influence of feedstock characteristics and cultivation conditions on protein production. Feedstock composition determines the availability of carbon, nitrogen, and other nutrients required for microbial growth, whereas cultivation parameters such as substrate loading and fermentation duration influence nutrient utilization and protein accumulation. Therefore, optimization of both feedstock characteristics and cultivation conditions is important for improving SCP production from food-processing wastes [29-31].

**Agricultural and agro-industrial by-products.** Agricultural and agro-industrial by-products are also attractive feedstocks for SCP production because they are generated continuously from agricultural and industrial activities. The substrates evaluated in this category included guava peel, cashew bagasse, and potato starch processing wastewater. Both solid-state fermentation and submerged fermentation were applied depending on the type of substrate. Guava peel and cashew bagasse were cultivated using solid-state fermentation at 30°C and 70% relative humidity with a substrate loading of 6% (w/v) [32]. In contrast, potato starch processing wastewater was cultivated in submerged fermentation using untreated wastewater and a 10% inoculum level. These differences highlight the influence of feedstock characteristics on the selection of cultivation systems and operating conditions for SCP production [33].

The findings suggest SCP production was influenced by both feedstock characteristics and cultivation conditions [28]. Among the evaluated substrates, potato starch processing wastewater achieved the highest crude protein content of 28.10%, followed by guava peel at 27.00% and cashew bagasse at 15.30%. This variation may reflect differences in nutrient availability among feedstocks as well as cultivation conditions, particularly fermentation mode and substrate loading, which influence substrate utilization and protein accumulation. Biomass yield and crude protein content also exhibited distinct production profiles, indicating that biomass formation and protein synthesis responded differently under the evaluated cultivation conditions. This difference reflects the distinct metabolic requirements for cell growth and protein synthesis, as biomass formation is largely driven by carbon utilization, whereas protein accumulation also depends on nitrogen assimilation and amino acid synthesis. Consequently, increased biomass formation does not necessarily result in a proportional increase in cellular protein content [32, 33].

**One-carbon feedstocks.** One-carbon feedstocks have gained increasing attention as alternative substrates for SCP production because they provide carbon sources that do not rely on conventional carbohydrate-rich materials [22]. Acetate and methanol were the one-carbon feedstocks evaluated in the studies summarized in Table 1. Acetate was cultivated using submerged fermentation at 25°C and pH 5.6 for 144 h, whereas methanol-based systems were mainly operated using fed-batch fermentation at temperatures between 28°C and 30°C [34]. Compared with waste-derived feedstocks, cultivation of one-carbon substrates generally involved tighter control of substrate feeding throughout the fermentation process. This was particularly evident in methanol-based systems, where fed-batch operation was consistently applied. These cultivation strategies enabled controlled substrate availability, supporting microbial growth and protein accumulation [16, 21, 22].

In particular, one-carbon feedstocks exhibited consistently high crude protein contents across the evaluated studies. Methanol-based cultivations produced crude protein contents ranging from 50.60% to 67.20% whereas acetate achieved 65.00% crude protein under submerged fermentation conditions [16, 21, 22]. These values were generally higher than those reported for food-processing wastes and agricultural by-products. The high protein contents therefore appear to reflect both the efficient utilization of one-carbon feedstocks and the controlled cultivation conditions, particularly substrate feeding in methanol-based systems. Moreover, biomass concentrations of up to 121.20 g DCW/L were reported, demonstrating the capacity of one-carbon substrates to support substantial microbial growth. However, biomass concentration and cellular protein content represent different production outcomes and should not be interpreted as directly proportional. Their responses depend on the combined effects of substrate utilization, strain characteristics, and cultivation conditions [16].

## Strategies for Enhancing Biomass and Protein Content in SCP

Several strategies have been developed to enhance single-cell protein (SCP) production in yeast, which can be broadly categorized into genetic engineering and evolutionary and process-based strategies.

### Genetic Engineering Strategies

Genetic engineering plays a central role in enhancing SCP production by enabling targeted modification of yeast metabolism and cellular functions. Metabolic engineering optimizes pathways for carbon and nitrogen assimilation, energy generation, and biosynthesis, improving substrate utilization and nutrient conversion efficiency, and thereby increasing biomass and protein yield [14, 35]. Additionally, genetic interventions can reprogram cellular structures and regulatory networks to direct resources more efficiently toward protein biosynthesis, which is essential for maximizing SCP production [7, 16]. Key strategies include: (i) modification of cell wall biogenesis and signaling pathways, (ii) metabolic and transporter engineering for improved substrate utilization, and (iii) engineering of nitrogen and amino acid metabolism.

Importantly, biomass formation and cellular protein accumulation represent distinct but interconnected outcomes of nutrient allocation. Assimilated carbon supports energy generation and cellular biomass formation, whereas nitrogen assimilation contributes directly to amino acid and protein biosynthesis. Consequently, strategies promoting biomass formation may produce different responses in cellular protein accumulation [17].

**Modification of cell wall biogenesis and signaling pathways.** Engineering the cell wall represents an effective genetic engineering strategy for enhancing SCP production by modulating cellular permeability, stress sensing, and metabolic flux [17]. Given that the yeast cell wall accounts for approximately 15–30% of cellular dry weight [36], its targeted modification can reduce carbon allocation to structural components and redirect metabolic resources toward protein biosynthesis [37]. Furthermore, the cell wall is a highly dynamic structure that responds to environmental stress through conserved signaling pathways, particularly the cell wall integrity (CWI) and high-osmolarity glycerol (HOG) pathways, which coordinate stress adaptation with metabolic regulation [17, 38].

Consistent with this, targeted engineering of cell wall biogenesis genes has been shown to significantly enhance protein accumulation in *S. cerevisiae*. For instance, quadruple deletion of *TIR4*, *SBE2*, *SBE22*, and *ECM25* increased protein content to 45.77 g/100 g DCW, representing a 21.61% improvement over the parental strain. In parallel, modification of key regulators in the HOG pathway, including *PBS2*, *HOG1*, *SHO1*, *STE11*, and *SSK2/SSK22*, further modulates stress responses and metabolic allocation, thereby influencing both biomass formation and protein synthesis. Notably, an optimized strain integrating both cell wall and HOG pathway engineering ultimately achieved a 32.6% increase in cellular protein content [17].

Similar strategies have been applied in *P. pastoris* for methanol-based SCP production. Comparative transcriptomics identified PAS_0305 as a key regulator of cell wall thickness. Deletion of this gene increased cell wall permeability and activated stress-response signaling, as indicated by trehalose accumulation. This perturbation triggered cross-talk between the HOG and CWI pathways via the Pbs2–Mkk module, leading to adaptive cell wall remodeling characterized by increased β-1,3-glucan and reduced chitin and mannose, thereby enhancing stress tolerance. Importantly, these signaling changes also drove metabolic reprogramming, redirecting carbon flux from methanol toward biomass and protein synthesis. Consequently, the engineered ΔPAS_0305 strain achieved a crude protein content of 67.21% and a methanol conversion rate of 0.46 g DCW/g [16].

Engineering cell wall biogenesis and signaling pathways also presents challenges due to the essential roles of these systems in maintaining cellular integrity and coordinating stress responses [39]. The CWI and HOG pathways regulate multiple interconnected cellular processes, making it important to consider how their modification affects cellular physiology beyond biomass and protein production. Understanding these regulatory interactions is therefore important for maintaining the robustness and stability of engineered yeast strains under industrial cultivation conditions.

**Metabolic and transporter engineering for enhanced substrate utilization.** Agricultural and food industry residues represent abundant and low-cost substrates for SCP production, offering a sustainable route to convert waste streams into high-value protein while mitigating environmental impacts. Feedstocks such as fruit residues, hemicellulosic biomass, and whey are widely explored due to their availability and nutrient content. [40]. However, their efficient utilization remains limited by substrate uptake and metabolic constraints. To address these challenges, metabolic engineering has been applied to expand substrate utilization pathways in yeast. In *S. cerevisiae*, heterologous expression of pathways for xylose (*XYL1*, *XYL2*, *XYL3*), arabinose (*LAD1*, *ALX1*), and galacturonic acid (*GAA1/gaaA*, *LGD1*, *GAA C*), combined with deletion of competing pathways (*PHO13*, *ALD6*), enables co-utilization of mixed sugars and improves carbon conversion efficiency [41]. In parallel, transporter engineering plays a critical role in enhancing substrate uptake. Optimization of membrane transport systems, including glucose (*HXT1*, *HXT7*), sucrose (*AGT1*), cellodextrin (*CDT-1*), xylose (Hxt family), and galactose (*GAL2*) transporters, further improves substrate assimilation and overall productivity [42].

In addition to substrate expansion, recent advances have focused on improving carbon utilization through CO_2_ recycling. Integration of a RuBisCO-based CO_2_-fixation pathway into a xylose-utilizing *S. cerevisiae* enabled incorporation of CO_2_ into central metabolism, as confirmed by flux balance and ^13^C-labeling analyses. Further enhancement through *PAN2* truncation increased protein content and amino acid levels under anaerobic conditions [43].

Despite their potential to improve substrate utilization, metabolic and transporter engineering require coordinated optimization of substrate uptake and intracellular metabolism. Enhanced transport does not necessarily lead to efficient cellular conversion, while excessive transporter expression or pathway modification may increase energy demand and metabolic burden, potentially affecting cellular fitness and protein accumulation [44]. Therefore, transporter engineering should be carefully balanced with cellular metabolic capacity to ensure efficient substrate utilization while maintaining biomass formation and protein accumulation.

**Engineering of nitrogen and amino acid metabolism.** Engineering of nitrogen metabolism has emerged as an effective strategy to enhance protein content in *P. pastoris*. In particular, overexpression of genes involved in ammonium assimilation, such as *GLN1* (glutamine synthase), has been shown to increase protein levels without compromising cell growth [22]. More recently, increasing attention has been directed toward improving protein quality through the production of amino acid-enriched SCP. For example, enrichment of branched-chain amino acids (BCAAs) has been achieved by integrating metabolic engineering with artificial intelligence. Overexpression of the key biosynthetic gene *Ilv3* enhanced endogenous BCAA production, while the PMPEPE (*P. pastoris* Mutation Predictor for Enhanced Protein Expression) model enabled the design of a BCAA-rich protein variant (M0504, 35% BCAA content). As a result, the engineered strain exhibited increased protein content and biomass during methanol-based fermentation, with BCAA levels reaching 9.8 mg/100 mg, representing a 37.1% increase over the wild type [21].

Although targeted engineering can increase protein content and enrich specific amino acids, nitrogen metabolism is tightly regulated, making it difficult to redirect nitrogen toward protein synthesis without affecting other cellular processes [45]. Changes in nitrogen assimilation or amino acid biosynthesis may also disturb metabolic balance and influence cell growth. Maintaining balanced nitrogen allocation remains an important consideration when engineering yeast for improved protein content and nutritional quality.

### Evolutionary and Process-Based Strategies

In addition to genetic engineering approaches, non-genetic-based strategies offer effective alternatives for improving SCP production, particularly in situations where regulatory restrictions or consumer concerns limit the use of genetically modified organisms. These approaches enhance microbial performance through physiological adaptation, process optimization, and careful strain selection, without direct modification of the genetic material.

**Co-culture strategies.** Beyond single-strain selection, co-culture strategies have been increasingly explored to enhance SCP production, particularly when utilizing complex waste substrates. For example, SCP production from potato starch processing wastewater has been improved through the co-cultivation of *C. utilis*, *Geotrichum candidum*, and *Candida tropicalis*. Under the optimized fermentation conditions, the system achieved an SCP yield of 3.06 g/L, a water-soluble protein content of 17.32%, and a chemical oxygen demand (COD) removal efficiency of 56.9% [33].

**Fermentation optimization.** Yield and productivity of SCP production are strongly influenced by culture medium composition and environmental conditions. Key parameters, including pH, temperature, incubation time, dissolved oxygen, aeration rate, and the availability of carbon and nitrogen sources, play critical roles in microbial growth and protein accumulation. These conditions may affect biomass formation and protein accumulation differently; therefore, cultivation conditions that maximize cell growth do not necessarily maximize cellular

protein content [46]. Consequently, systematic optimization of these factors is essential to maximize SCP production efficiency. To facilitate this process, design of experiments (DoE) approaches, particularly factorial design and response surface methodology (RSM), are widely applied due to their ability to simultaneously evaluate multiple variables and identify optimal operating conditions [47]. For example, RSM has been successfully applied to enhance SCP production in *Y. lipolytica* grown on biofuel waste, where key variables such as temperature and pH were optimized. Under these conditions, protein concentration increased by 44% compared to the initial value [48].

Fermentation optimization can improve SCP yield and productivity by providing suitable conditions for yeast growth and protein accumulation. However, process performance can change during scale-up due to differences in mixing, oxygen transfer, and nutrient availability between laboratory and larger bioreactors [49]. Further optimization at larger scales is thus needed to maintain stable SCP production under industrial conditions.

**Random mutagenesis.** Random mutagenesis is a classical strategy for generating microbial strains with desirable phenotypes by introducing genetic diversity without prior knowledge of metabolic bottlenecks. This approach can be achieved using physical mutagens, such as UV radiation, X-rays, and particle radiation, or chemical mutagens, followed by effective screening methods to identify improved strains [50]. UV-induced mutagenesis has been applied to generate a *S. cerevisiae* mutant library, which was subsequently screened over multiple rounds to identify strains with improved protein-related traits. The selected mutant (#1021) exhibited a 31% increase in amino acid content and a 23% increase in concentration compared to the parental strain. Further analysis linked these improvements to a single nucleotide polymorphism in *PAN2*, which encodes a catalytic subunit of the poly(A)-nuclease (PAN) deadenylation complex [18].[Fig F1]

In addition to conventional approaches, advanced irradiation-based mutagenesis techniques such as atmospheric and room temperature plasma (ARTP) have emerged as efficient alternatives for strain improvement. These methods are often combined with high-throughput screening strategies to enable rapid evaluation of large mutant libraries. Fluorescence-based systems, including microfluidics-assisted fluorescence-activated droplet sorting (FADS), allow efficient detection of complex traits such as protein synthesis capacity. The integration of ARTP mutagenesis with such screening platforms has enabled the identification of *S. cerevisiae* mutants with enhanced protein production, achieving increases of approximately 10% in protein content and up to 18% in yield [51]. Nevertheless, screening remains a practical challenge in random mutagenesis because large numbers of mutants must be evaluated to identify strains with desirable phenotypes [52]. This challenge becomes particularly relevant when screening for complex traits such as protein production.

**Adaptive laboratory evolution (ALE).** ALE is a process in which microorganisms undergo successive selection under defined conditions, leading to the enrichment of more fit genotypes and a cumulative improvement in overall fitness [53]. This strategy has been widely applied to enhance key traits relevant to SCP production, including substrate utilization efficiency, stress tolerance, and protein accumulation. ALE has been employed in *P. pastoris* to overcome limitations in methanol utilization efficiency and tolerance to elevated temperatures (33°C). Long-term evolution resulted in reduced carbon loss, likely due to decreased detoxification of intracellular formaldehyde via the dissimilation pathway, as well as cell wall remodeling that contributed to improved thermal stress resistance, as revealed by transcriptomic and phenotypic analyses [22].

Moreover, ALE can be combined with mutagenesis to increase genetic diversity and accelerate the evolution of desired phenotypes. In *S. cerevisiae*, the integration of ALE with ARTP mutagenesis and high-throughput screening enabled the development of strains with significantly enhanced protein content. This approach generated four mutant libraries (~200,000 cells) under protein synthesis inhibitor pressure, leading to the isolation of a high-protein strain that achieved 70.3 g/100 g dry cell weight in a 45 L fed-batch bioreactor (ethanol < 1.5 g/L), representing an 18.5% improvement. Transcriptomic analysis further revealed that the increased protein content was associated with downregulation of cell cycle- and meiosis-related genes [19]. sALE is effective for improving complex traits, but the long evolution period can make strain development time-consuming. Accelerating the selection process while maintaining stable phenotypes could improve the overall efficiency of this approach [54].

## Application of SCP

**Fig. 2:**
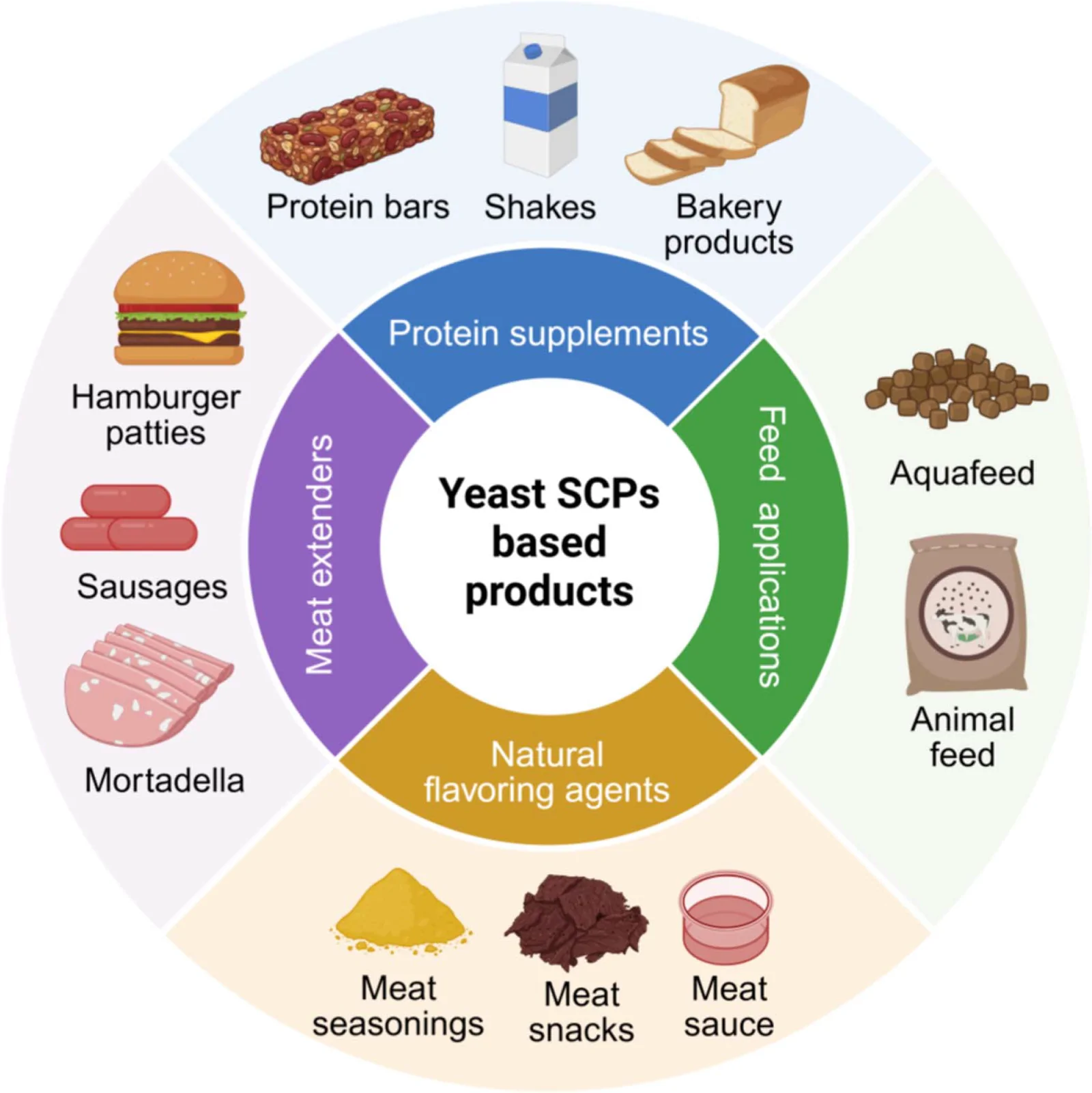
Applications of yeast-based single-cell protein (SCP). Yeast-based SCP can be utilized in a wide range of food and feed applications, including protein supplements (protein bars, shakes, and bakery products), feed ingredients for livestock and aquaculture, natural flavoring agents (meat seasonings, meat snacks, and meat sauces), and meat extenders in processed meat products such as hamburger patties, sausages, and mortadella. These diverse applications highlight the nutritional, functional, and industrial potential of yeast-derived proteins in sustainable food and feed systems. (Created with BioRender.com).

Yeast proteins are considered a safe and high-quality protein source with considerable potential for food applications. Their favorable nutritional profile and functional properties have supported their incorporation into a variety of food products. In addition to providing protein, yeast-derived ingredients can contribute valuable bioactive compounds and technological functionalities that enhance product quality. The current applications of yeast proteins in food systems are summarized in Fig. 2.

### Protein Supplements

Yeast-based SCP has gained increasing attention as a protein supplement because of its high protein content and good nutritional quality. In addition to providing essential amino acids, yeast proteins can be produced sustainably using microbial fermentation and renewable substrates. The European Food Safety Authority (EFSA) has approved proteins derived from *Y. lipolytica* as a dietary supplement, highlighting their safety and nutritional value for human consumption [55]. Furthermore, yeast proteins have low allergenic potential and can serve as an alternative protein source for consumers seeking sustainable and nutritionally balanced diets [56].

The favorable nutritional and functional properties of yeast proteins have supported their application in a variety of food products. Yeast-derived proteins have been incorporated into bakery products, cookies, snacks, protein bars, and nutritional supplements to increase protein content. In addition, their high glutamic acid content contributes desirable flavor characteristics, making them suitable ingredients for processed products. Yeast proteins can also be used in dairy-alternative products, providing additional options for consumers with lactose intolerance or specific dietary preferences. These applications demonstrate the potential of yeast-based SCP as a versatile ingredient in food formulations [57].

### Meat Extenders

Meat extenders represent one of the most promising food applications of yeast-based SCP. Yeast biomass has been incorporated into processed meat products such as mortadella, sausages, and hamburger patties due to its favorable emulsifying properties and oil-holding capacity. These functional characteristics contribute to improved texture, structural integrity, and dimensional stability, while also reducing cooking losses during thermal processing. In addition, the incorporation of yeast-derived ingredients can enhance water retention within the meat matrix, resulting in products with improved juiciness and tenderness [57].

Yeast protein can also serve as a valuable nutritional ingredient in meat products. Burger patties formulated with natural yeast protein have been reported to exhibit nutritional compositions comparable to conventional formulations in terms of moisture, protein, lipid, and carbohydrate contents. Furthermore, the high water-holding capacity of yeast proteins contributes to improved product stability and overall quality. The presence of glutamic acid and other flavor-active compounds may also enhance the sensory characteristics of meat products. These attributes demonstrate the potential of yeast-based SCP as a sustainable and multifunctional ingredient for processed meat applications [58].

### Natural Flavoring Agents

Natural flavoring agents represent another important application of yeast-based SCP in the food industry. During fermentation, yeasts produce a variety of flavor-active compounds that can contribute desirable sensory characteristics to food products. In addition, yeast extracts contain proteins, peptides, amino acids, and nucleotides that serve as important sources of non-volatile flavor compounds [59]. These components are widely recognized for their ability to impart umami and kokumi tastes, thereby enhancing overall flavor intensity and complexity. As a result, yeast-derived ingredients have been incorporated into a range of food products as natural flavor enhancers and, in some cases, as partial salt replacers in low-sodium formulations [60].

The flavor-enhancing properties of yeast-derived ingredients have supported their use in various processed foods and seasonings. Yeast extracts are commonly utilized in meat snacks, meat sauces, condiments, and other savory products to improve flavor depth and palatability. Furthermore, yeast-derived flavor compounds can help mask undesirable odors and contribute to a more balanced and harmonious flavor profile. Beyond sensory improvement, the natural origin and safety of yeast-based ingredients have further increased their attractiveness as clean-label flavoring agents. These characteristics highlight the potential of yeast-based SCP and yeast-derived products as multifunctional ingredients in food formulation and flavor development [61].

### Feed Applications

Animal and aquaculture feed represent some of the most established applications of yeast-based SCP due to its high protein content, balanced amino acid profile, and favorable digestibility. In aquaculture, yeast protein has been incorporated into fish and shrimp feeds as a sustainable alternative to conventional protein ingredients, supporting growth performance and feed utilization [62]. In addition, yeast-derived proteins have been utilized in pet food formulations for dogs and cats, where their high nutritional quality and hypoallergenic properties make them attractive ingredients. The growing interest in yeast-based SCP for feed applications is also driven by the need to identify alternative protein sources that can reduce dependence on conventional feed ingredients while maintaining nutritional value [63].

Beyond aquaculture and companion animals, yeast protein has also demonstrated considerable potential in livestock nutrition. In poultry production, supplementation with yeast protein has been associated with improved growth performance and feed efficiency in both broilers and laying hens. Similarly, in dairy cattle, yeast-derived proteins have been reported to enhance milk yield, milk protein content, and rumen function. Positive effects have also been observed in swine production, where the high digestibility of yeast protein supports growth and feed conversion efficiency during the weaning and growing phases. Collectively, these findings highlight the versatility of yeast-based SCP as a high-quality protein ingredient for a broad range of animal feed applications [64].

## Conclusion and Future Perspectives

Recent advances in metabolic engineering, adaptive laboratory evolution, random mutagenesis, and fermentation optimization have significantly improved yeast-based single-cell protein (SCP) production from renewable substrates. Nevertheless, substantial variations in SCP production are still observed among food-processing wastes, agricultural by-products, and one-carbon feedstocks due to differences in substrate composition and nutrient availability [6, 65]. Future research should therefore focus on developing robust yeast strains and optimized cultivation strategies capable of efficiently utilizing diverse renewable feedstocks while maintaining stable protein production performance. Such improvements will further support waste valorization and strengthen the role of yeast-based SCP in sustainable circular bioeconomy systems.

Further improvement in yeast-based SCP production requires a better understanding of the metabolic and regulatory mechanisms that determine substrate utilization, biomass formation, and protein accumulation. These traits are closely interconnected, and their responses can vary depending on the yeast strain, feedstock composition, and cultivation conditions. System-level analyses integrating genomic, proteomic, and metabolic data provide a comprehensive view of these cellular responses and help identify metabolic bottlenecks and regulatory mechanisms that limit efficient conversion of renewable substrates into protein-rich biomass [8].

In this regard, artificial intelligence (AI) offers a promising approach for analyzing complex biological datasets and accelerating yeast SCP development. AI-assisted approaches can support metabolic pathway modeling, predict the effects of genetic modifications, and identify combinations of engineering targets associated with improved substrate utilization and protein production [7]. Integration with synthetic biology and metabolic engineering can further guide strain modification toward multiple SCP-related traits, including efficient substrate utilization, improved stress tolerance, increased protein content, and desirable amino acid composition [66]. Incorporating AI-assisted prediction into Design-Build-Test-Learn (DBTL) workflows could further enable iterative strain and process optimization [67]. Combined with high throughput screening and fermentation optimization, these approach could accelerate the development of robust yeast strains and scalable production processes, supporting the industrial application of yeast-based SCP in sustainable food and feed system [68, 69].

## Figures and Tables

**Fig. 1 F1:**
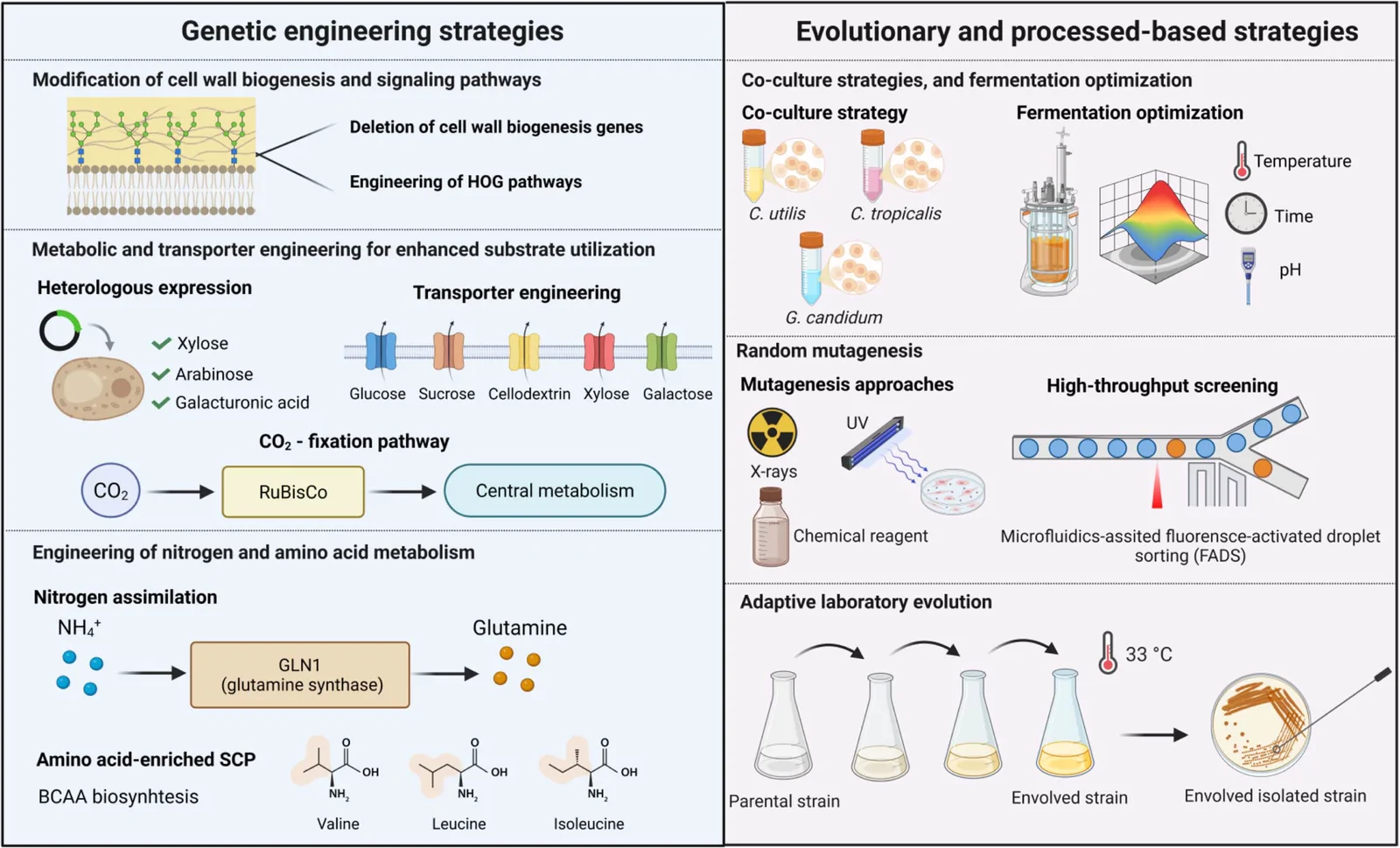
Strategies for enhancing biomass and protein content in SCP. Several strategies have been developed to enhance single-cell protein (SCP) production in yeast, which can be broadly categorized into genetic engineering and evolutionary and process-based strategies (Created with BioRender.com).

**Table 1:** Overview of yeast species, fermentation conditions, biomass concentration, and protein production from various renewable feedstocks.